# A Performance Benchmark for Dedicated Short-Range Communications and LTE-Based Cellular-V2X in the Context of Vehicle-to-Infrastructure Communication and Urban Scenarios

**DOI:** 10.3390/s21155095

**Published:** 2021-07-28

**Authors:** Tibor Petrov, Lukas Sevcik, Peter Pocta, Milan Dado

**Affiliations:** 1 Department of International Research Projects—ERAdiate+, University of Zilina, Univerzitna 1, 010 26 Zilina, Slovakia; 2 Department of Multimedia and Information-Communication Technology, Faculty of Electrical Engineering and Information Technology, University of Zilina, Univerzitna 1, 010 26 Zilina, Slovakia; lukas.sevcik@uniza.sk (L.S.); peter.pocta@fel.uniza.sk (P.P.); milan.dado@uniza.sk (M.D.)

**Keywords:** dedicated short-range communications, cellular-V2X, vehicle-to-infrastructure communication, packet delivery ratio, end-to-end delay

## Abstract

For more than a decade, communication systems based on the IEEE 802.11p technology—often referred to as Dedicated Short-Range Communications (DSRC)—have been considered a de facto industry standard for Vehicle-to-Infrastructure (V2I) communication. The technology, however, is often criticized for its poor scalability, its suboptimal channel access method, and the need to install additional roadside infrastructure. In 3GPP Release 14, the functionality of existing cellular networks has been extended to support V2X use cases in an attempt to address the well-known drawbacks of the DSRC. In this paper, we present a complex simulation study in order to benchmark both technologies in a V2I communication context and an urban scenario. In particular, we compare the DSRC, LTE in the infrastructural mode (LTE-I), and LTE Device-to-Device (LTE-D2D) mode 3 in terms of the average end-to-end delay and Packet Delivery Ratio (PDR) under varying communication conditions achieved through the variation of the communication perimeter, message generation frequency, and road traffic intensity. The obtained results are put into the context of the networking and connectivity requirements of the most popular V2I C-ITS services. The simulation results indicate that only the DSRC technology is able to support the investigated V2I communication scenarios without any major limitations, achieving an average end-to-end delay of less than 100 milliseconds and a PDR above 96% in all of the investigated simulation scenarios. The LTE-I is applicable for the most of the low-frequency V2I services in a limited communication perimeter (<600 m) and for lower traffic intensities (<1000 vehicles per hour), achieving a delay pf less than 500 milliseconds and a PDR of up to 92%. The LTE-D2D in mode 3 achieves too great of an end-to-end delay (above 1000 milliseconds) and a PDR below 72%; thus, it is not suitable for the V2I services under consideration in a perimeter larger than 200 m. Moreover, the LTE-D2D mode 3 is very sensitive to the distance between the transmitter and its serving eNodeB, which heavily impacts the PDR achieved.

## 1. Introduction

In the last years, the interest in vehicular communications has rapidly increased. The main reason behind this is the fact that the establishment of ad hoc communication networks between vehicles, as well as with the corresponding infrastructure—referred to as vehicle-to-everything (V2X) communication in the literature—is expected to considerably reduce the number of accidents and to improve traffic management. The benefits of this paradigm, which is called the Intelligent Transportation Systems (ITS) in the literature, are so important that research in this area is considered as a strategic topic for transport authorities in the European Union and most developed countries.

When it comes to V2X communication, there are currently two competing technologies, i.e., Dedicated Short-Range Communications (DSRC) and cellular-V2X (C-V2X). The former is based on the IEEE 802.11-2012 standard, which was later superseded by the IEEE 802.11-2016 standard. On the other hand, the C-V2X was described in the specifications of 4G LTE Release 14. The DSRC technology was mainly developed to enable collision prevention applications [1]. It is widely regarded to be reliable, adequate, and, most importantly, immediately available. Moreover, it is also almost patent-free, rather easy to implement, and financially rather cheap. On the other hand, the C-V2X is claimed to have a wider application range in areas such as entertainment, traffic data, navigation, and, most notably, autonomous driving. In comparison to the DSRC, it can leverage existing infrastructure and can cover larger areas with less infrastructural equipment, thus possibly reducing the capital and operational expenditures for the infrastructure’s owners [2], but unfortunately not for its users [3], unless a no-SIM operational approach is adopted.

Some work has been carried out to separately evaluate the performance of C-V2X—i.e., LTE-V2X—and IEEE 802.11p, as well as on a comparative basis. In [4], intensive data analytics on V2V performance and characterization of the V2V channel based on real-world urban DSRC traces were presented, and a reliable context-aware beaconing strategy called CoBe was proposed to enhance the broadcast reliability by coping with harsh NLoS conditions. Moreover, the authors of [3] dealt with the application-level performance of safety-related communications over IEEE 802.11p in the Infrastructure-to-Vehicle (I2V) context and presented the first outcomes resulting from several short- and medium-range field tests. They studied five different performance metrics that were relevant at the network, transport, and application levels. Their figures and trends were examined against six variables, i.e., distance, transmission power, antenna quality, congestion, interference, and speed. On the other hand, the performance of LTE-V2X in the context of the I2V and V2V communication was investigated by using simulations in [5]. When it comes to the benchmarking of C-V2X and IEEE 802.11p in terms of their performance, the following studies focused on this issue: [6,7,8,9,10,11,12]. In [6], the authors benchmarked LTE-V2X and IEEE 802.11p in terms of the average packet reception ratio in typical urban and freeway scenarios and the V2V context. The DSRC and LTE-V2X were compared from the perspective of the packet delivery success rate in different transport scenarios, i.e., cities and highways, in [7]; again, this was only in the V2V context. In [8], ITS-G5 and LTE-V2X (mode 3) were benchmarked in terms of the end-to-end delay and radio frequency conditions by measuring the signal to interference plus noise ratio and reference signal received power in two scenarios, i.e., the effects of realistic data traffic on an ITS alert service and the impact of handover on an ITS safety service; again, this was only in the V2V context. In [9], the benchmarking was performed in an urban micro-cell highway scenario and the V2V context from the perspective of the packet reception ratio and transmitter–receiver distance. In [10,11], the both communication systems were investigated in the V2V context from perspective of the end-to-end delay, packet delivery ratio, and throughput and from the perspective of the average packet reception ratio, respectively. In more detail, the authors of [10] evaluated the performance of the IEEE 802.11p standard and LTE technology for vehicular networks under various network conditions and parameter values in terms of reliability, delay, and scalability. For [11], the evaluation indicated that LTE-based alternatives, including the LTE multicast and LTE sidelink, provide better performance than the IEEE 802.11p in terms of the reliable communication range reached in all of the studied scenarios. Finally, the corresponding technologies were compared from the point of view of periodic and aperiodic messages of constant and variable sizes in [12]. In summary, as can be clearly seen from the papers listed above, most studies of the performance have focused on the V2V communication context when dealing with the technologies’ performance or benchmarking them in terms of their performance.

To the best of our knowledge, there is no work dealing specifically with the benchmarking of C-V2X (neither 4G-based nor 5G-based) and DSRC in the context of V2I communication. It is worth noting here that V2I communication is particularly important when it comes to traffic management. Therefore, we decided to focus on this issue in this paper. So, we benchmarked the DSRC and LTE-based C-V2X in the infrastructural mode, as well as in the device-to-device mode 3, in terms of the Packet Delivery Ratio (PDR) and end-to-end delay in the context of V2I communication and urban scenarios from the perspective of traffic intensity, message generation frequency, and communication perimeter. The obtained results are put into the context of the networking and connectivity requirements of popular V2I C-ITS services in terms of the PDR and end-to-end delay.

The remainder of this paper is organized as follows. Section 2 describes the test setup and simulation environment. In Section 3, the experimental results are presented and discussed. Section 4 provides the final conclusions.

## 2. Setup and Simulation Environment

To compare the performance of the three selected networking technologies—i.e., DSRC, LTE-infrastructural, and LTE Device-to-Device mode 3—in a vehicular environment, we prepared a federated telco-traffic simulation experiment. To realistically simulate the vehicular mobility, a traffic microsimulation was run in the Simulation of Urban Mobility (SUMO) traffic simulator [13] with a realistic model of the road infrastructure in the city of Zilina (Slovakia). The SUMO simulation served as a mobility generator for communication nodes in the OMNeT++ discrete event network simulator [14], where the corresponding communication protocols were simulated. The positions of all vehicles were synchronized between the two simulators during the simulation runtime via a bi-directional Traffic Control Interface (TraCI) [13], a part of the SUMO traffic simulator. The communication protocols of the DSRC network were modeled using the Veins simulation framework [15]. The protocol stack of the LTE network was modeled using the SimuLTE simulation framework [16]. The higher communication layers were modeled using the INET simulation framework [17].

### 2.1. Simulation Scenarios

**Figure 1:**
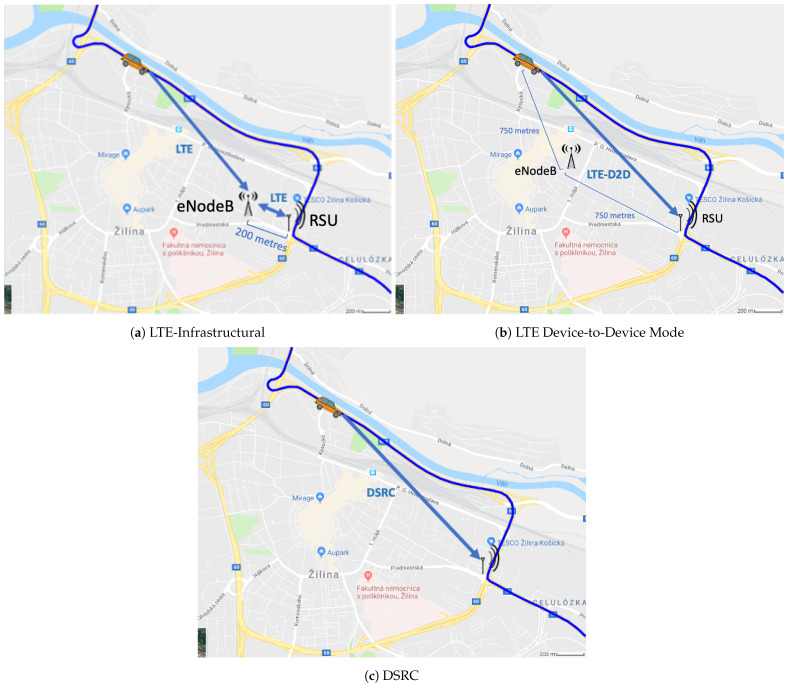
Visualization of the simulation scenarios.

In the simulation study presented here, we measured the average Packet Delivery Ratio (PDR) and average end-to-end delay of Vehicle-to-Infrastructure (V2I) communication in an urban scenario in which vehicles communicated with a Roadside Unit (RSU) located at an intersection in the city of Zilina (averaged over all of the communication sessions realized in the corresponding simulation runs); see Figure 1 for more details. In the first simulation scenario, the V2I communication was carried out by an LTE network consisting of an eNodeB placed 200 m from the RSU at the intersection. All of the vehicles, as well as the RSU, were wirelessly connected to the same cell by using the Uu interface and E-UTRA Operating Band 65 [18]; see Figure 1a for more details. The network topology of the second simulation scenario (see Figure 1b for more details) was similar, except for the fact that the communication in this case was carried out with the LTE Device-to-Device (D2D) Mode 3 [19]. The payload packets were transmitted directly from the vehicles to the RSU. So, the eNodeB just oversaw the network and managed a scheduling process for the communication resources, but did not take part in the transmission of the payload. In this case, because the eNodeB did not provide data connectivity to the RSU, it was moved closer to the road segment from which the vehicle flow in order to facilitate the signaling of the resource selection to the transmitting vehicles more reliably. The communication in the third scenario (see Figure 1c for more details) was based on the DSRC technology. The vehicles and RSU formed an ad hoc network that included the PHY and MAC layers, as defined in IEEE 802.11p [20].

### 2.2. Simulation Settings

To explore each technology’s sensitivity to traffic volume/intensity, we varied the traffic intensity in 6 steps, ranging from 250 to 1500 vehicles per hour. The values of the traffic intensity were set according to the official data from a traffic survey that was conducted for the General Traffic Plan of the City of Zilina. A well-established microscopic car-following model based on [21] was used to model the inter-vehicular spacings. The vehicle flow generated was a Poisson process where λ was equal to the respective traffic intensity value. For the sake of the comparability of the results, the traffic flows—down to the level of individual vehicle dynamics—were kept identical between the simulation scenarios for each given level of traffic intensity. The message generation frequency was varied in 5 steps from 2 to 10 Hz to characterize different ITS services; see [22] for more details. To study the impact of distance on the measured parameters, i.e., the PDR and end-to-end delay, the communication perimeter was varied in 7 steps ranging from 200 to 1400 m. All of the simulation variables and their respective values are summarized in Table 1.

The simulation parameters applied are detailed in Table 2. The vehicles periodically transmitted unacknowledged messages with a fixed length to the RSU, i.e., emulating the Cooperative Awareness Message (CAM) service over the UDP protocol. To prevent the possible distortion of the simulation results as a result of a change in the traffic flow, the RSU did not influence the intersection control in any way upon the successful receipt of a message. Apart from the periodic (CAM) messages, there was also event-triggered communication traffic present in the typical C-ITS system. Represented by Decentralized Environmental Notification Messages (DENMs) in Europe, these event-triggered messages are more likely to be generated with increasing traffic intensity. As their amount is highly stochastic and very specific to the scenario and application, we did not consider them in this study. It is worth noting here that frequent dissemination of DENMs can further congest the communication channel and induce collisions on the Medium Access Layer (MAC), especially in the case of DSRC.

Each simulation run simulated 10 min of traffic with an additional 100 s to represent the initialization period used to populate the transport network with vehicles. Over this initialization period, the target communication performance indicators, i.e., the PDR and end-to-end delay, were not evaluated.

The technology-specific communication parameters of LTE and DSRC used for the simulation experiments are presented in Table 3 and Table 4, respectively.

It is worth noting here that all of the remaining system parameters that were not specifically listed in the tables above were set to the default values recommended by the simulator developers and clearly described/specified in the corresponding documentation.

## 3. The Experimental Results

**Figure 2:**
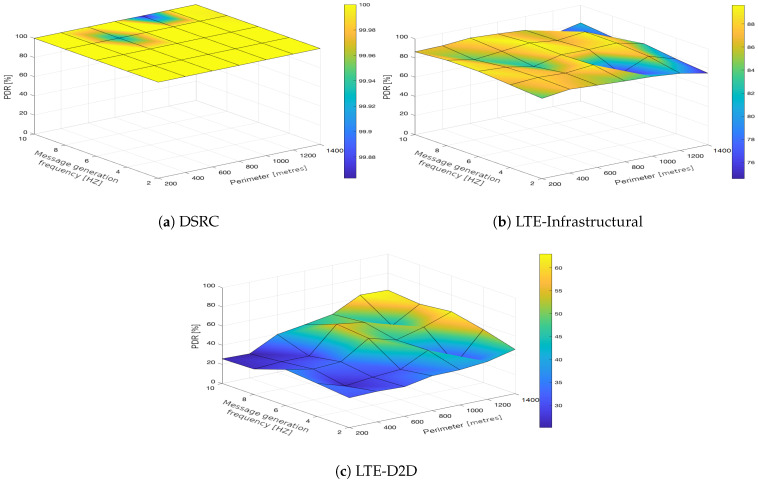
Packet delivery ratio obtained for the traffic intensity of 250 vehicles per hour.

**Figure 3:**
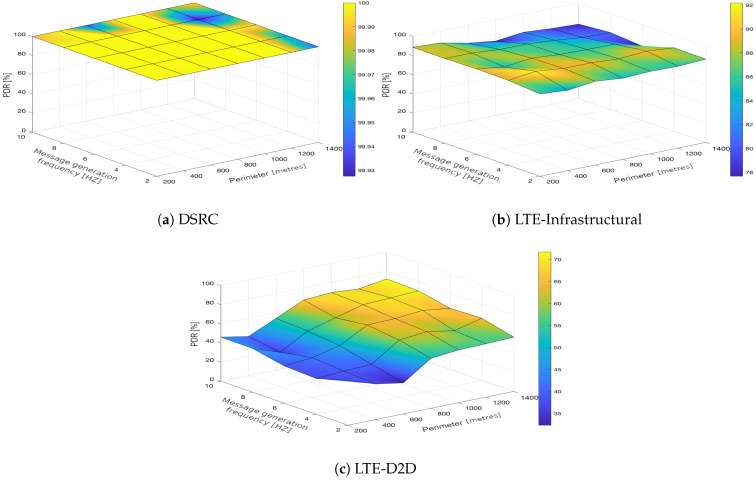
Packet delivery ratio obtained for the traffic intensity of 500 vehicles per hour.

**Figure 4:**
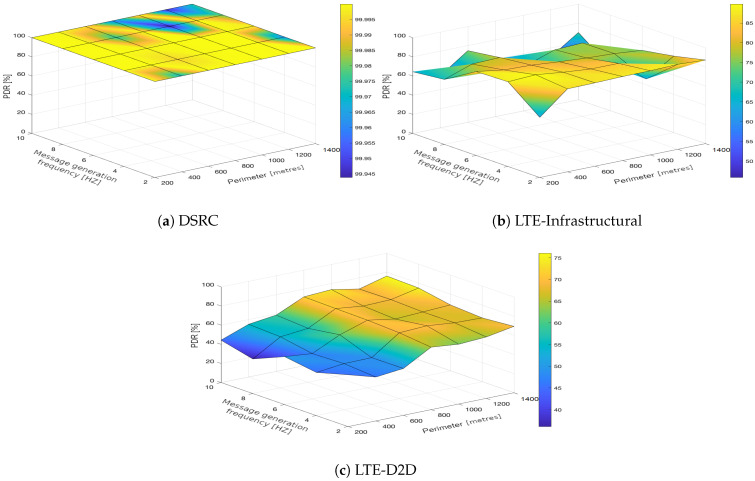
Packet delivery ratio obtained for the traffic intensity of 750 vehicles per hour.

**Figure 5:**
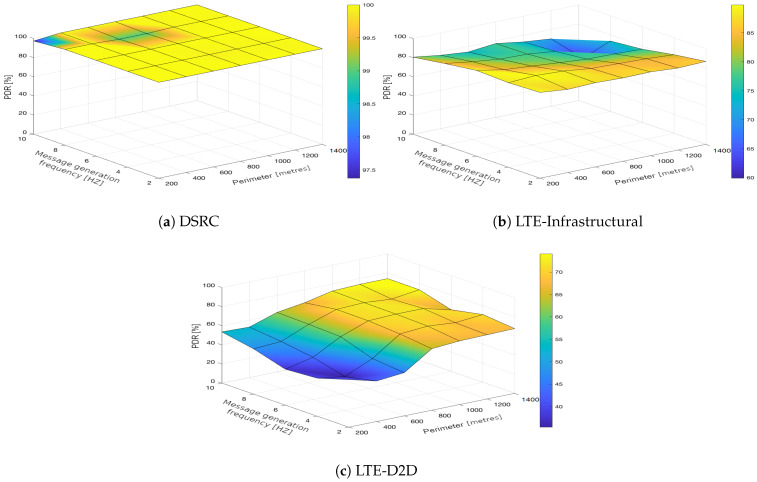
Packet delivery ratio obtained for the traffic intensity of 1000 vehicles per hour.

**Figure 6:**
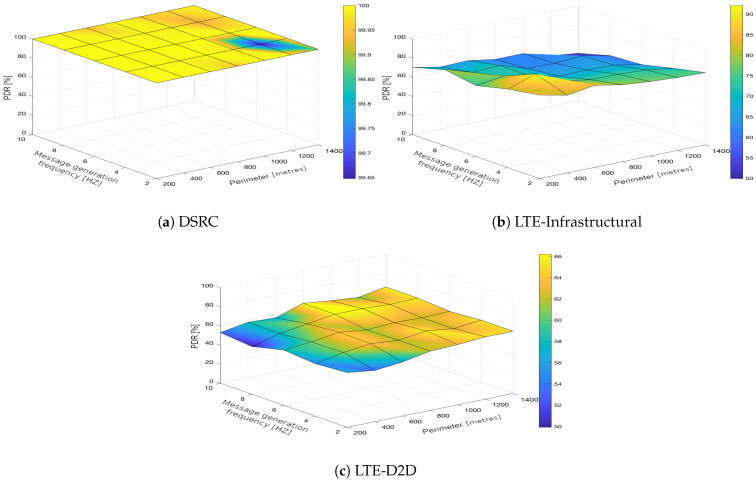
Packet delivery ratio obtained for the traffic intensity of 1250 vehicles per hour.

**Figure 7:**
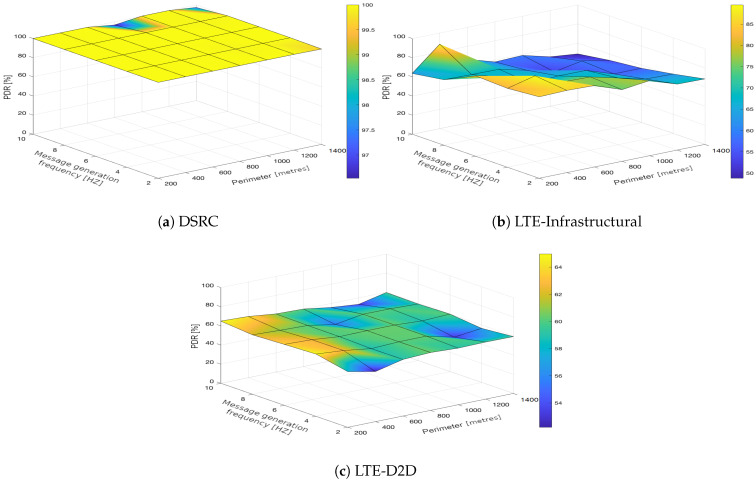
Packet delivery ratio obtained for the traffic intensity of 1500 vehicles per hour.

**Figure 8:**
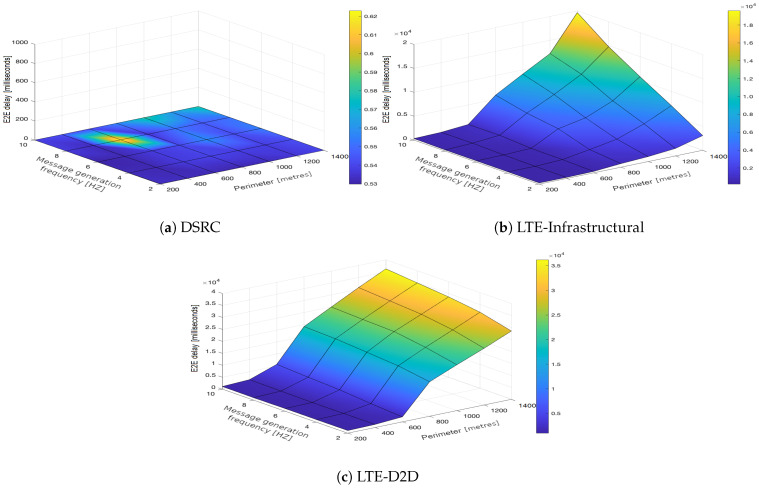
End-to-end delay obtained for the traffic intensity of 250 vehicles per hour.

**Figure 9:**
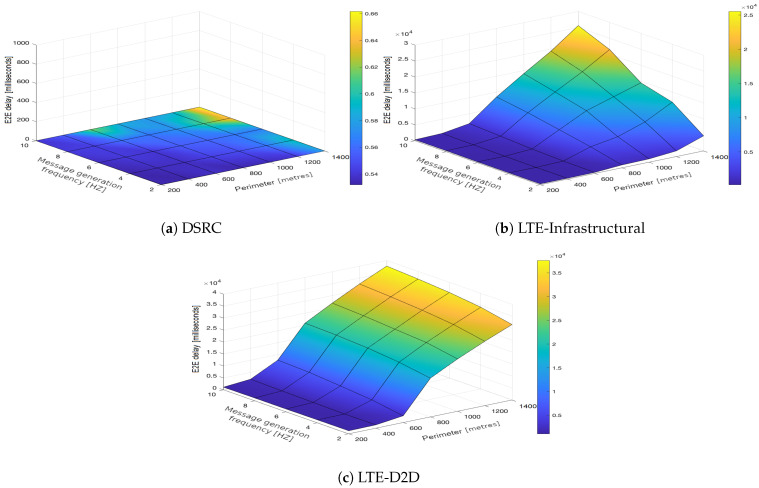
End-to-end delay obtained for the traffic intensity of 500 vehicles per hour.

**Figure 10:**
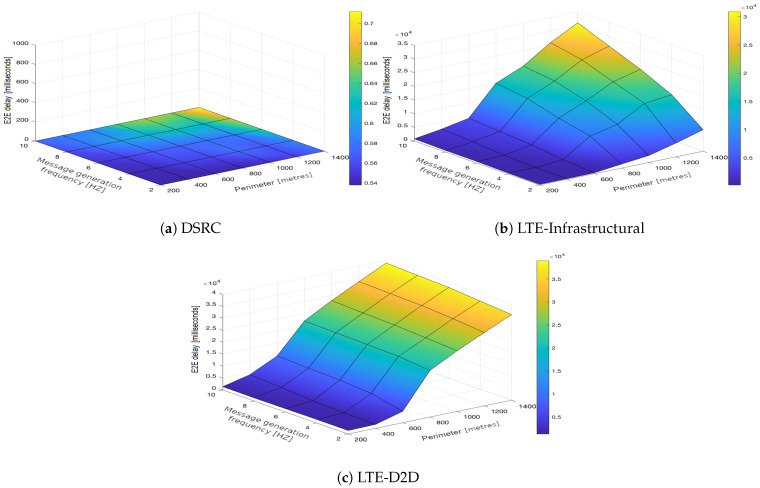
End-to-end delay obtained for the traffic intensity of 750 vehicles per hour.

**Figure 11:**
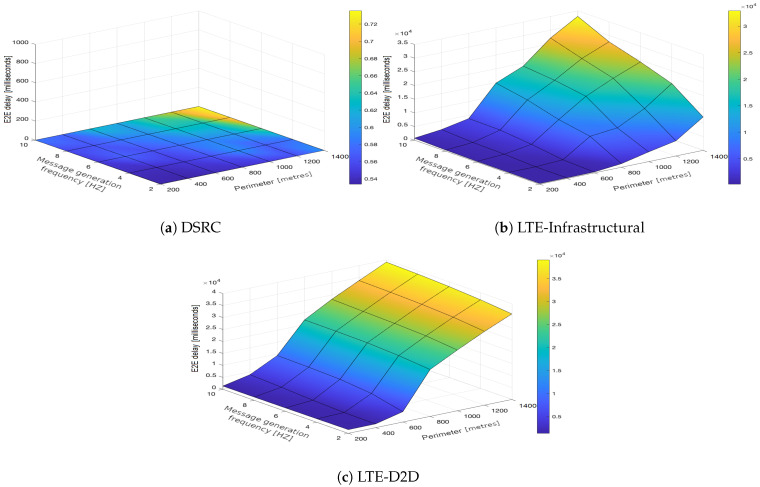
End-to-end delay obtained for the traffic intensity of 1000 vehicles per hour.

**Figure 12:**
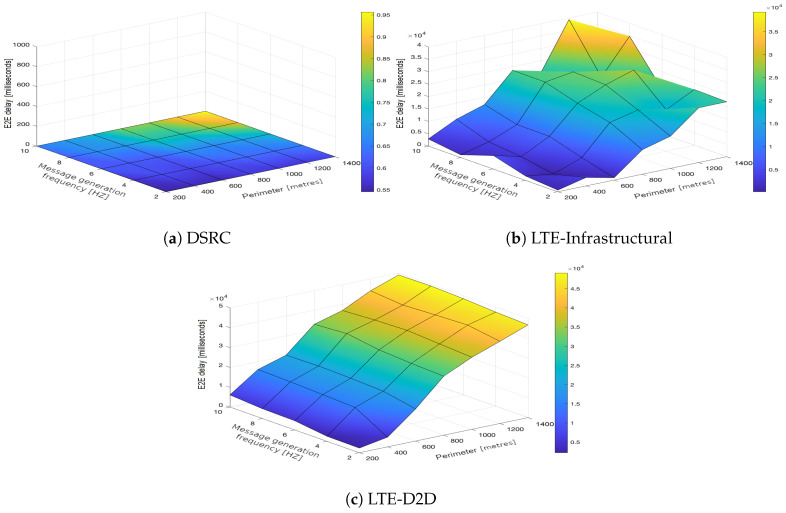
End-to-end delay obtained for the traffic intensity of 1250 vehicles per hour.

**Figure 13:**
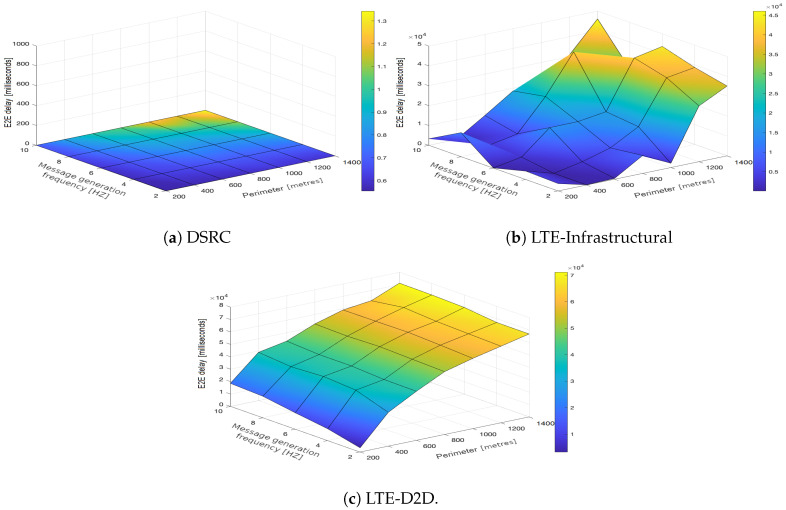
End-to-end delay obtained for the traffic intensity of 1500 vehicles per hour.

In the upcoming subsections, the results are presented and compared. In particular, the PDR results are presented in Section 3.1, graphically illustrated in Figure 2, Figure 3, Figure 4, Figure 5, Figure 6 and Figure 7, and the end-to-end delay results are described in Section 3.2, graphically illustrated in Figure 8, Figure 9, Figure 10, Figure 11, Figure 12 and Figure 13. In both cases, we benchmarked the DSRC, LTE-Infrastructural, and LTE-D2D (mode 3) technologies with different traffic intensity values from the perspective of the communication perimeter and message generation frequency. Finally, the results obtained were put into the context of the networking and connectivity requirements of the most popular V2I services.

### 3.1. Packet Delivery Ratio

The DSRC was confirmed to have a high reliability in a dynamic network topology. The PDR had a high rating with minimal deviations. We could say that the trend was more or less constant, as it was very close to the maximum PDR value (Figure 2a, Figure 3a, Figure 4a, Figure 5a, Figure 6a and Figure 7a); that is, for the most part, the communication perimeter and the message generation frequency did not play a role in this case. When we compare the results obtained for the DSRC and LTE technologies, we can conclude that the PDR values obtained were much worse in both LTE scenarios, i.e., the LTE-Infrastructural (Figure 2b, Figure 3b, Figure 4b, Figure 5b, Figure 6b and Figure 7b), and LTE-D2D mode (Figure 2c, Figure 3c, Figure 4c, Figure 5c, Figure 6c and Figure 7c). The best PDR (around 92%) was obtained for the LTE-Infrastructural mode. With the increase in the message generation frequency for the two lowest traffic intensities, the PDR was a bit higher. On the other hand, the PDR decreased with higher values of the traffic intensity. The communication perimeter had a rather minor impact when it came to the lowest traffic intensities. The impact was more severe for the higher ones, but was still rather negligible in comparison to the LTE-D2D scenario. It achieved the worst results when the high message generation frequency and the largest perimeter were combined.

In the LTE-D2D case, the behavior was quite different (Figure 2c, Figure 3c, Figure 4c, Figure 5c, Figure 6c and Figure 7c). The increased value of the perimeter caused a higher PDR. This was also the case for the higher message generation frequency, except with the communication perimeter of 200 m. The best results were achieved for the combination of the greater perimeter and higher message generation frequency. This sounds rather unintuitive at first glance; however, the reason for this behavior lies in the topology of the communication network. On one hand, with a decreasing perimeter, the communicating vehicle is closer to the RSU. On the other hand, its distance from the serving eNodeB increases, which results in less reliable resource scheduling for the transmitting vehicles, and collisions start to occur. Hence, the simulation results suggest that in the case of the LTE-D2D mode 3 communication, the distance between the transmitter and the eNodeB might have an even greater impact on the PDR than the distance between the transmitter and receiver.

Moreover, a comparison of the LTE scenarios is rather interesting, as the opposite trends are nicely visible in the graphs, i.e., the communication perimeter played a rather important role in the case of LTE-D2D, which was not the case when it came to the LTE-Infrastructural mode. Moreover, the PDR in the LTE-D2D scenario was very dependent on the distance of the transmitter from the serving eNodeB. This was probably due to the fact that the resource scheduling was unreliable over long distances, and collisions occurred.

We can conclude that the traffic intensities of 250 and 500 vehicles per hour in the LTE-Infrastructural mode offered the best results (Figure 2b and Figure 3b). It is worth noting here that rather low values of the PDR were reported for the higher traffic intensities. Moreover, the LTE-D2D mode achieved only around 70% of the PDR, even in the case of the highest perimeter and with higher numbers of generated messages.

### 3.2. Average End-to-End Message Latency

It is worth reiterating here that the presented values represent the average delay, i.e., averaged over all communication sessions realized in the corresponding simulation runs. Similarly to with the PDR, the DSRC also obtained much better results when it came to the end-to-end delay (Figure 8a, Figure 9a, Figure 10a, Figure 11a, Figure 12a and Figure 13a). With increasing values of the message generation frequency and communication perimeter, the delay also increased. Naturally, the lowest delay values were reported for the lowest values of the perimeter and message generation frequency. Moreover, decreasing traffic intensity led to better values of the delay, especially for higher values of the perimeter and message generation frequency. For both LTE modes, i.e., LTE-Infrastructural (Figure 8b, Figure 9b, Figure 10b, Figure 11b, Figure 12b and Figure 13b) and LTE-D2D (Figure 8c, Figure 9c, Figure 10c, Figure 11c, Figure 12c and Figure 13c), the behavior was the same as that reported for the DSRC. So, the delay values increased with the increase in the perimeter or frequency of the generated messages. Regarding the traffic intensity, the trend was again the same as that reported for the DSRC. Please note that the values of the average end-to-end delay were approximately four orders of magnitude lower in the case of the DSRC than for both LTE-based technologies.

Generally, we can conclude that neither mode of LTE was suitable for perimeters greater than 600 m. A lower frequency of message generation (two or four) could also be used for perimeters of 800–1000 m in the infrastructural mode. The D2D mode was applicable only for very short distances/perimeters. With greater perimeters, the traffic intensity began to play a more important role. It is worth noting here that the absolute value of the delay was significantly reduced in the LTE communications when the infrastructural mode was deployed. However, with the increasing number of communicating vehicles, it was easy to see how the end-to-end delay started to fluctuate as the PDR grew and HARQ retransmissions began to occur, as illustrated in Figure 12b and Figure 13b.

This effect was observed when there were more than 90 simultaneously communicating vehicles within the same cell, and its significance grew with the increasing traffic intensity. The PDR in these cases was as low as 50%.

Putting the obtained results into the context of the networking and connectivity requirements of the most popular V2I services [22], the DSRC was able to fulfill the latency requirements for all the types of V2I services in all of the investigated urban scenarios. The LTE in the infrastructural mode was able to partially fulfill the requirements within a limited communication perimeter (<600 m) for the services that required less frequent updates (1–2 Hz) and that had less stringent latency requirements (<500 milliseconds). V2I services that require low-latency communication (<100 milliseconds) combined with less frequent updates (1–2 Hz) could be served by the LTE-Infrastructural technology within a limited perimeter (<600 m) and for traffic intensities of up to 1000 vehicles per hour. The LTE in the D2D mode 3 was not able to serve any of the most popular V2I services in the selected use case. Moreover, the simulation results suggest that in all of the investigated scenarios, only the DSRC was able to support the V2I services with the most stringent requirements in terms of latency and message generation frequency. Table 5 summarizes the compliance of each investigated communication technology in the context of the selected urban scenario with the networking and connectivity requirements of the most popular V2I services.

## 4. Conclusions

Unlike a conventional network infrastructure, the possibility of achieving guaranteed high-level QoS is almost unattainable in the case of Vehicular Ad hoc Networks (VANETs). The reason is the absence of a consistent infrastructure and the environment’s dynamism, which involves frequent and sudden changes in the topology. Typically, QoS routing technologies use a fixed and reliable line connection. Minimal emphasis is placed on a possible renewal. However, connections (routing paths) created in a dynamic infrastructure are influenced by speed, position, or delay. Due to the potentially high speeds achieved by vehicles, the emphasis is placed on the fastest possible way of transmitting warning messages. To achieve reliable real-time communication, the importance of high-performance routing protocols in VANETs is very high.

Based on this knowledge, this study benchmarked the DSRC and LTE-based C-V2X technologies in terms of the PDR and end-to-end delay in the V2I context and urban scenarios. Regarding the LTE-based C-V2X, two technologies, i.e., LTE-Infrastructural and LTE Device-to-Device mode 3, were investigated.

Using a fixed-topology transport network, we varied the traffic intensity, message generation frequency, and communication perimeter. Beyond the evident fact that the end-to-end latency tended to grow with the increase in the communication perimeter, message generation frequency, and traffic intensity, the simulation results showed major differences between the investigated technologies in terms of their sensitivity to changes in these simulation variables. Among the three investigated technologies, the DSRC performed well in the widest range of variations of the parameters. The LTE in the infrastructural mode was sensitive to increases in the message generation frequency, thus placing a higher signalization burden on the network, while the latency in LTE-D2D mode 3 grew rapidly with the increasing communication perimeter. In terms of the PDR, the DSRC yielded a stable performance with a PDR of greater than 96% under all of the conditions investigated. The LTE in the infrastructural mode achieved a PDR of between 50 and 92%, and the highest PDR values were achieved when both the communication perimeter and message generation frequency were low. Finally, the LTE-D2D in mode 3 constantly achieved a PDR of less than 72%. When the distance between the transmitter and the resource-allocating eNodeB was highest, the PDR of the LTE-D2D dropped to values as low as 25%.

Furthermore, we put these results into the context of the most popular V2I services and their networking and connectivity requirements. Our results indicate that only the DRSC is fully able to support the V2I services with varying demands on the communication latency and message generation frequency in the investigated urban scenario. The LTE in the infrastructural mode is able to support low-frequency applications for communication perimeters of up to 600 m and even low-latency applications for traffic intensities below 1000 vehicles per hour. The LTE in D2D mode 3 is not suitable for longer-range V2I communication.

However, it is worth noting here that the situation may change considerably with the deployment of 5G-based Vehicle-to-Everything (5G V2X) communication systems. These 5G V2X systems are expected to bring major improvements in link quality and throughput, as well as reduced latency. As a follow up, our aim is to benchmark 5G V2X against DSRC and LTE-V2X in a V2I scenario once the appropriate simulation framework for the corresponding simulation stack becomes available.

## Figures and Tables

**Table 1:** Simulation variables.

Parameter	Value
Traffic intensity	250, 500, 750, 1000, 1250, 1500 vehicles per hour
Message generation frequency	2, 4, 6, 8, 10 Hz
Communication perimeter	200, 400, 600, 800, 1000, 1200, 1400 m

**Table 2:** Simulation parameters.

Parameter	Value
Application protocol	CAM-like periodic fixed-length message exchange service
Transport protocol	UDP
Message length	300 bytes (including a security header)
Simulation length	600 s
Number of repetitions	10

**Table 3:** LTE-specific parameters.

Parameter	Value
Frequency band	2100 MHz
Channel bandwidth	10 MHz
Transmit power	40 dBm
Max. HARQ Retransmission	3
eNodeB height	25 metres
Thermal noise	−104.5 dBm
eNodeB antenna gain	18 dBi
UE antenna gain	0 dBi
eNodeB noise figure	5 dB
UE noise figure	7 dB
Cable loss	2 dB
Number of fading paths (JAKES)	6

**Table 4:** DSRC-specific parameters.

Parameter	Value
Carrier frequency	5900 MHz
Channel bandwidth	10 MHz
Data rate	6 Mbps
Transmit power	20 dBm
Path loss model	Two Ray Interference
TX antenna gain	0 dBi
RX antenna gain	0 dBi

**Table 5:** Networking and connectivity requirements of the most popular V2I services and their fulfillment by the investigated communication technologies in the context of the selected urban scenario. Adapted and updated from [22].

V2I Service	DSRC	LTE-Infrastructural	LTE-D2D
**Low-frequency (1–2 Hz), low-latency (<100 milliseconds) services**			
Slow and stationary vehicle warning	✓	✓– up to 600 m & 1000 vehicles per hour	✕
Weather condition warnings	✓	✓– up to 600 m & 1000 vehicles per hour	✕
Intersection management	✓	✓– up to 600 m & 1000 vehicles per hour	✕
**Low-frequency (1–2 Hz), high-latency (<500 milliseconds) services**			
Point of interest notification	✓	✓– up to 600 m	✕
Local electronic commerce	✓	✓– up to 600 m	✕
Media upload	✓	✓– up to 600 m	✕
Map updates	✓	✓– up to 600 m	✕
Cooperative flexible lane change	✓	✓– up to 600 m	✕
**High-frequency (10 Hz), low-latency (<100 milliseconds) services**			
Electronic emergency brake light	✓	✕	✕
Emergency vehicle approaching	✓	✕	✕

## Data Availability

The simulation data is available upon request.
